# KIAA1199 Correlates With Tumor Microenvironment and Immune Infiltration in Lung Adenocarcinoma as a Potential Prognostic Biomarker

**DOI:** 10.3389/pore.2022.1610754

**Published:** 2022-11-07

**Authors:** Xiaoju Shen, Xiaocheng Mo, Weidan Tan, Xiaoxiang Mo, Li Li, Fei Yu, Jingchuan He, Zhihua Deng, Shangping Xing, Zhiquan Chen, Jie Yang

**Affiliations:** ^1^ Department of Pharmacology, School of Pharmacy, Guangxi Medical University, Nanning, China; ^2^ Department of Pharmacy, The First Affiliated Hospital of Guangxi Medical University, Nanning, China; ^3^ Department of Pharmacology, Guangxi Institute of Chinese Medicine and Pharmaceutical Science, Nanning, China; ^4^ Guangxi Key Laboratory of Bioactive Molecules Research and Evaluation, School of Pharmacy, Guangxi Medical University, Nanning, China

**Keywords:** biomarker, prognosis, lung adenocarcinoma, immune infiltration, KIAA1199

## Abstract

**Background:** KIAA1199 has been considered a key regulator of carcinogenesis. However, the relationship between KIAA1199 and immune infiltrates, as well as its prognostic value in lung adenocarcinoma (LUAD) remains unclear.

**Methods:** The expression of KIAA1199 and its influence on tumor prognosis were analyzed using a series of databases, comprising TIMER, GEPIA, UALCAN, LCE, Prognoscan and Kaplan-Meier Plotter. Further, immunohistochemistry (IHC), western blot (WB) and receiver operating characteristic (ROC) curve analyses were performed to verify our findings. The cBioPortal was used to investigate the genomic alterations of KIAA1199. Prediction of candidate microRNA (miRNAs) and transcription factor (TF) targeting KIAA1199, as well as GO and KEGG analyses, were performed based on LinkedOmics. TIMER and TISIDB databases were used to explore the relationship between KIAA1199 and tumor immune infiltration.

**Results:** High expression of KIAA1199 was identified in LUAD and Lung squamous cell carcinoma (LUSC) patients. High expression of KIAA1199 indicated a worse prognosis in LUAD patients. The results of IHC and WB analyses showed that the expression level of KIAA1199 in tumor tissues was higher than that in adjacent tissues. GO and KEGG analyses indicated KIAA1199 was mainly involved in extracellular matrix (ECM)-receptor interaction and extracellular matrix structure constituent. KIAA1199 was positively correlated with infiltrating levels of CD4^+^ T cells, macrophages, neutrophil cells, dendritic cells, and showed positive relationship with immune marker subsets expression of a variety of immunosuppressive cells.

**Conclusion:** High expression of KIAA1199 predicts a poor prognosis of LUAD patients. KIAA1199 might exert its carcinogenic role in the tumor microenvironment *via* participating in the extracellular matrix formation and regulating the infiltration of immune cells in LUAD. The results indicate that KIAA1199 might be a novel biomarker for evaluating prognosis and immune cell infiltration in LUAD.

## Introduction

Lung cancer is one of the most common malignant tumors, and remains the leading cause of cancer-related deaths worldwide [1, 2]. Non-small cell lung cancer (NSCLC) accounts for 85% of all lung cancer cases. LUAD and LUSC are the two major histological subtypes in NSCLC [3]. Most patients with LUAD were diagnosed at the advanced stages due to late onset of clinical symptoms and inadequate screening methods, contributing to a low 5- year survival rate [1]. Treatments for LUAD usually include surgery, chemotherapy, molecular targeted therapy, immunotherapy and radiotherapy or combination therapy [4], of which advancements in targeted therapies and immunotherapies have achieved prominent success [5–7]. However, most patients will ultimately suffer disease progression and resistance to the first-line treatment regimen [8, 9]. The pathogenesis of LUAD is extremely complex, which is related to genetic mutations of cancer cells, changes in molecular characteristics, and interactions with tumor microenvironment [9–11]. Thus, novel and sensitive biomarkers for prognosis as well as immunotherapy targets for patients with LUAD are urgently needed.

KIAA1199, also known as cell migration inducing protein (CEMIP) or hyaluronan binding protein (HYBID), was reported to cause non-syndromic hearing loss and depolymerize hyaluronic acid (HA) [12, 13]. In recent years, well-documented studies have demonstrated that KIAA1199 plays a role in promoting malignant progression and predicts poor prognosis of cancer patients, including gastric cancer [14–16], pancreatic cancer [17], colorectal cancer [18, 19], prostate cancer [20, 21] and so on. Studies have shown that KIAA1199 can promote sorafenib tolerance and the metastasis of hepatocellular carcinoma [22], facilitate extracellular matrix-detached prostate cancer cell survival by inhibiting ferroptosis [23] and accelerate metastasis in other tumors [18–20]. In particular, recent studies indicate that KIAA1199 may show immune regulatory function to support malignant progression and metastasis through upregulation of CCL/CXCL cytokines for neutrophils recruitment [24, 25]. KIAA1199 expression is increased in NSCLC and closely related to invasion and migration of NSCLC cells [26, 27]. Nevertheless, biological functions and mechanisms of KIAA1199 in LUAD need to be further explored. Moreover, the relationship between KIAA1199 and tumor immune infiltration in LUAD has yet to be investigated.

In this study, public databases were used to perform a series of bioinformatics analyses to determine KIAA1199 expression and correlations between its expression and clinical outcomes in LUAD and LUSC patients. Regulatory network and genomic alterations of KIAA1199 were analyzed. Function and pathway enrichment analyses were performed to explore the biological function and underlying molecular mechanisms of KIAA1199 in LUAD. Additionally, the relationship between KIAA1199 expression and local immune cells in LUAD tumors was investigated. In a nutshell, our study will help to deepen the understanding of the intrinsic relationship and molecular mechanisms of KIAA1199 in LUAD.

## Materials and Methods

### KIAA1199 Gene Expression Analysis in LUAD and LUSC

The differential expression of KIAA1199 between tumor tissues of LUAD and LUSC and their corresponding adjacent normal tissues was evaluated by the “Gene_DE Module” of TIMER2.0 and GEPIA databases. TIMER2.0 is a webserver (http://timer.cistrome.org/), which contains 10,897 samples of 32 cancer types from The Cancer Genome Atlas (TCGA) [28]. GEPIA (http://gepia.cancer-pku.cn/) is a web tool for cancer and normal gene-expression profiling and interactive analyses based on TCGA and The Genotype-Tissue Expression (GTEx) data [29].

KIAA1199 expression in different pathological stages (stage I ∼ IV), nodal metastasis stage and other clinical features of LUAD and LUSC was obtained based on UALCAN database. UALCAN database (http://ualcan.path.uab.edu) [30] is an interactive web portal for analysis of TCGA RNA-seq data and clinicopathological characteristics of the patients from different cancer types.

### Survival Prognosis Analysis of KIAA1199 in LUAD and LUSC

The Kaplan–Meier plotter (https://kmplot.com/analysis/), which includes data from the Gene Expression Omnibus (GEO), TCGA and European Genome-phenome Atlas (EGA), is publicly available to assess the effect of various genes on patients survival of certain cancer types, including breast, ovarian, lung and gastric cancer [31]. PrognoScan (http://dna00.bio.kyutech.ac.jp/PrognoScan/index.html) is capable of assessing the prognostic value of genes by meta analyzing a large collection of published cancer microarray data [32]. The Kaplan–Meier plotter database and PrognoScan were used to analyze the correlations between KIAA1199 expression and patient survival in LUAD and LUSC. The hazard ratio (HR) with 95% confidence interval (95% CI) and logarithmic rank *p*-value (*p* < 0.05 is considered to be significant) were calculated.

Lung Cancer Explorer (LCE) database (http://lce.biohpc.swmed.edu/lungcancer/) [33] is a lung cancer-specific database housing expression data and clinical data. Based on the meta-analysis module in LCE database, we validated the prognostic role as well as the expression level of KIAA1199 in LUAD and LUSC. The association in each dataset was evaluated by Cox hazard models. Hazard ratios (HRs) and log-rank *p*-values were calculated.

### Genetic Alteration Analysis of KIAA1199 in LUAD

We analyzed the alteration frequency of KIAA1199 in LUAD based on data from “MSKCC 2020, MSKCC 2021, MSK 2021, Nat Genet 2020 and TCGA,” then summarized mutated site information of the KIAA1199 gene. The information based on the “Cancer Type Summary” and “Mutations” modes was obtained from the cBioPortal database (https://www.cbioportal.org), which is a comprehensive network resource that has been used to analyze the information on genetic alterations in various cancer genomic datasets [34].

### Function and Pathway Enrichment Analysis of KIAA1199 in LUAD

LinkedOmics (http://linkedomics.org/login.php) [35] is a web platform which contains multiple sets of data from 32 types of cancer within the TCGA. The LinkFinder module was used to identify genes co-expressed with KIAA1199. The correlation was tested by the Pearson test. GO analyses, including CC (cellular component), BP (biological process), MF (molecular function), KEGG pathway, miRNA targets, and TFs (transcription factors), were performed through the LinkInterpreter module.

### The Analysis of the Relationship Between KIAA1199 and Immunity in LUAD

TISIDB (http://cis.hku.hk/TISIDB/) [36] is a comprehensive database for conducting tumor and immune system interaction analysis that integrates multiple types of high-throughput data. The correlations among KIAA1199 expression and tumor-infiltrating lymphocytes (TILs), chemokines as well as chemokine receptors of LUAD were comprehensively investigated through TISIDB.

TIMER (https://cistrome.shinyapps.io/timer/) [28] is a comprehensive resource which allows to evaluate the abundance of the tumor infiltrates immune cells (TIICs) and to analyze the infiltration of immune cells in tumor tissues. The correlations among KIAA1199 expression and tumor purity as well as the abundance of six types of TIICs including B cells, CD4^+^ T cells, CD8^+^ T cells, neutrophils, macrophages and dendritic cells were investigated in LUAD using the gene module of TIMER. The Spearman method was used to determine the correlation coefficient.

### Patient Samples Collection and Examination

Seventy-two pairs of paraffin‐embedded tissues of LUAD and their matched tumor-adjacent tissues and 12 pairs of fresh LUAD tissues were obtained from the Affiliated Tumor Hospital of Guangxi Medical University. This study was approved by Ethic Committee of Guangxi Medical University (No.029, 2018) in accordance with the principles of the Declaration of Helsinki [37].

The expression of KIAA1199 in 12 pairs of fresh LUAD tumor tissues and their matched tumor-adjacent tissue was detected by WB. The procedure of WB assay followed the description in our previous report [38, 39]. The primary antibodies against KIAA1199 (1:800, Proteintech, China), β-Actin (1:2,000, ProteinTech, China) and secondary antibody, anti-rabbit IgG (DyLight™ 800 4X PEG Conjugate, CST, US) were used in this study. Protein bands signals were detected and visualized through infrared fluorescence imaging system Odyssey (LI-COR, United States), and analyzed by ImageJ Plus software.

The expression of KIAA1199 in seventy-two pairs of paraffin‐embedded tissues of LUAD patients and their matched tumor-adjacent tissues was detected by IHC, conducted following the procedure described below. The sections from paraffin‐embedded tissues were collected and dewaxed with xylene, hydrated with a gradient of ethyl alcohol. Antigen retrieval was performed in 0.01 M sodium citrate (pH 6) for 5 min with 115°C and high pressure (∼70 kPa). Then the sections were slowly cooled to room temperature. Samples were blocked with 3% hydrogen peroxide for 15 min and 5% BSA for 30 min at room temperature, and then probed overnight at 4°C with primary antibody: KIAA1199/CEMIP (21129-1-AP, Proteintech, 1:200), then recovered at 37°C for 1 h. After washing, the slides were incubated with the biotin-labeled goat anti-mouse/rabbit IgG and horseradish peroxidase-labeled *streptomyces* ovalbumin working solution (ZSGB-BIO, Beijing, China) for 15 min at room temperature, respectively. DAB Substrate Kit (ZSGB-BIO, Beijing, China) were used for staining in this study. All images were evaluated under optical microscopy at ×200 magnification. Four representative fields separated over the tumor tissue were randomly selected for quantitative analysis of histological staining using ImageJ Plus.

### Statistical Analysis

Statistical analysis carried out by online tools was automatically calculated and the *p*-value, or log rank *p*-value, or nominal *p*-value < 0.05 was considered as statistically significant. SPSS 20.0 (Chicago, IL, United States) and GraphPad Prism 8 software (La Jolla, CA, United States) were applied to the following statistical analyses. Non-paired Student’s t test was used to analyze the expression differences between 72 pairs of paraffin-embedded LUAD tissues and matched adjacent tissues, and between groups with and without metastasis. Non-parametric test was used for 12 pairs of fresh LUAD tissues. The relationships between 72 LUAD patients’ survival time and KIAA1199 protein expression as well as patients’ clinicopathologic factors were analyzed by Non-paired Student’s t test. The prognostic value was determined by multivariate Cox regression analysis. The cutoff value of KIAA1199 expression was selected by ROC curve and Youden’s index.

## Results

### The KIAA1199 Expression Level in LUAD and LUSC

**FIGURE 1:**
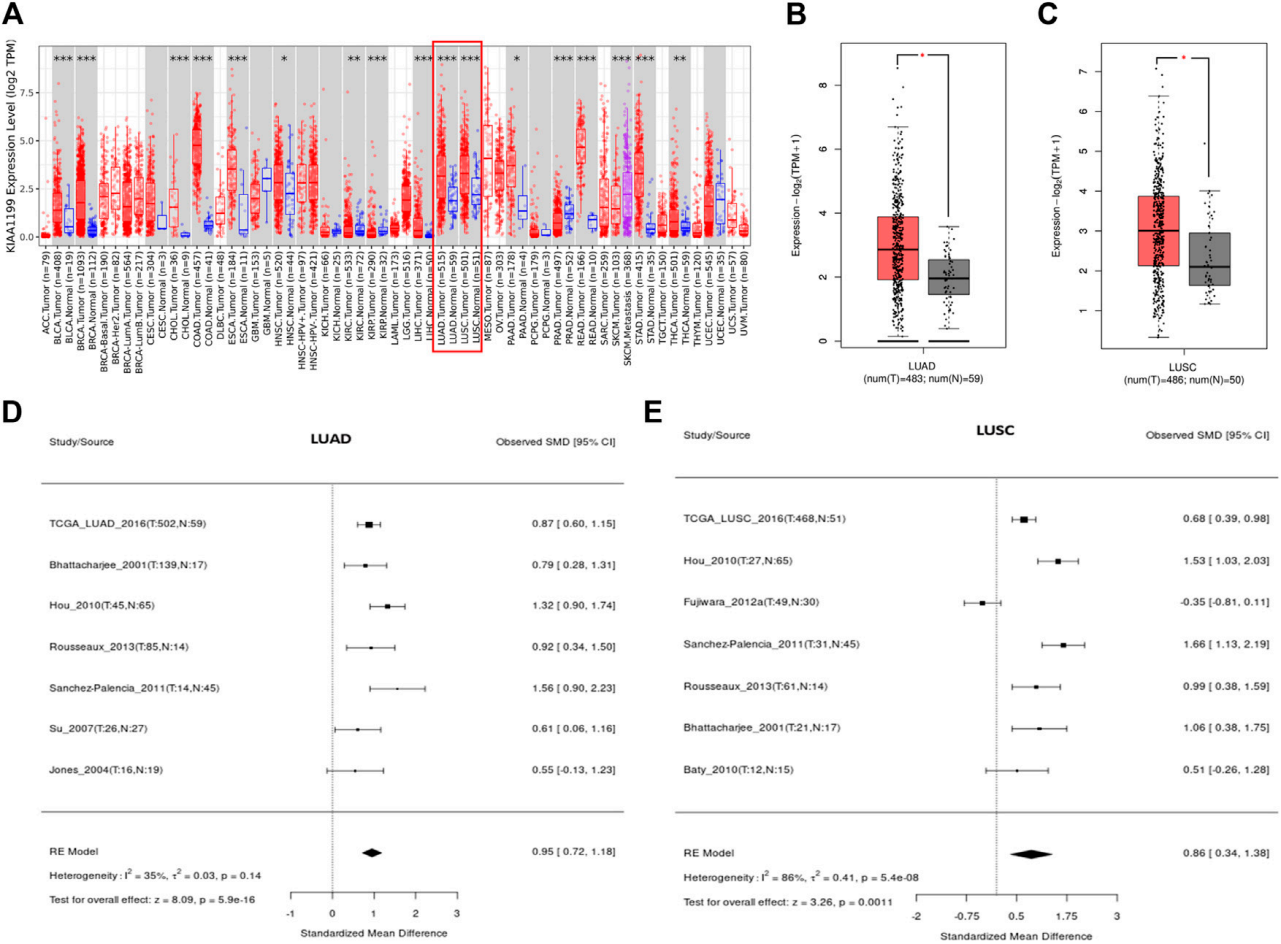
The expression level of KIAA1199 in different cancers. **(A)** The KIAA1199 expression level in various cancer tissues and their corresponding normal tissues were analyzed using the TIMER database. **(B,C)** The comparisons of RNA-seq expression levels in LUAD tissue (*n* = 483), LUSC tissue (*n* = 486) and normal control tissue (*n* = 59 for LUAD and *n* = 50 for LUSC). **(D,E)** The meta-analysis result of KIAA1199 mRNA expression in LUAD and LUSC based on LCE database. *p < 0.05; **p < 0.01; ***p < 0.001.

**FIGURE 2:**
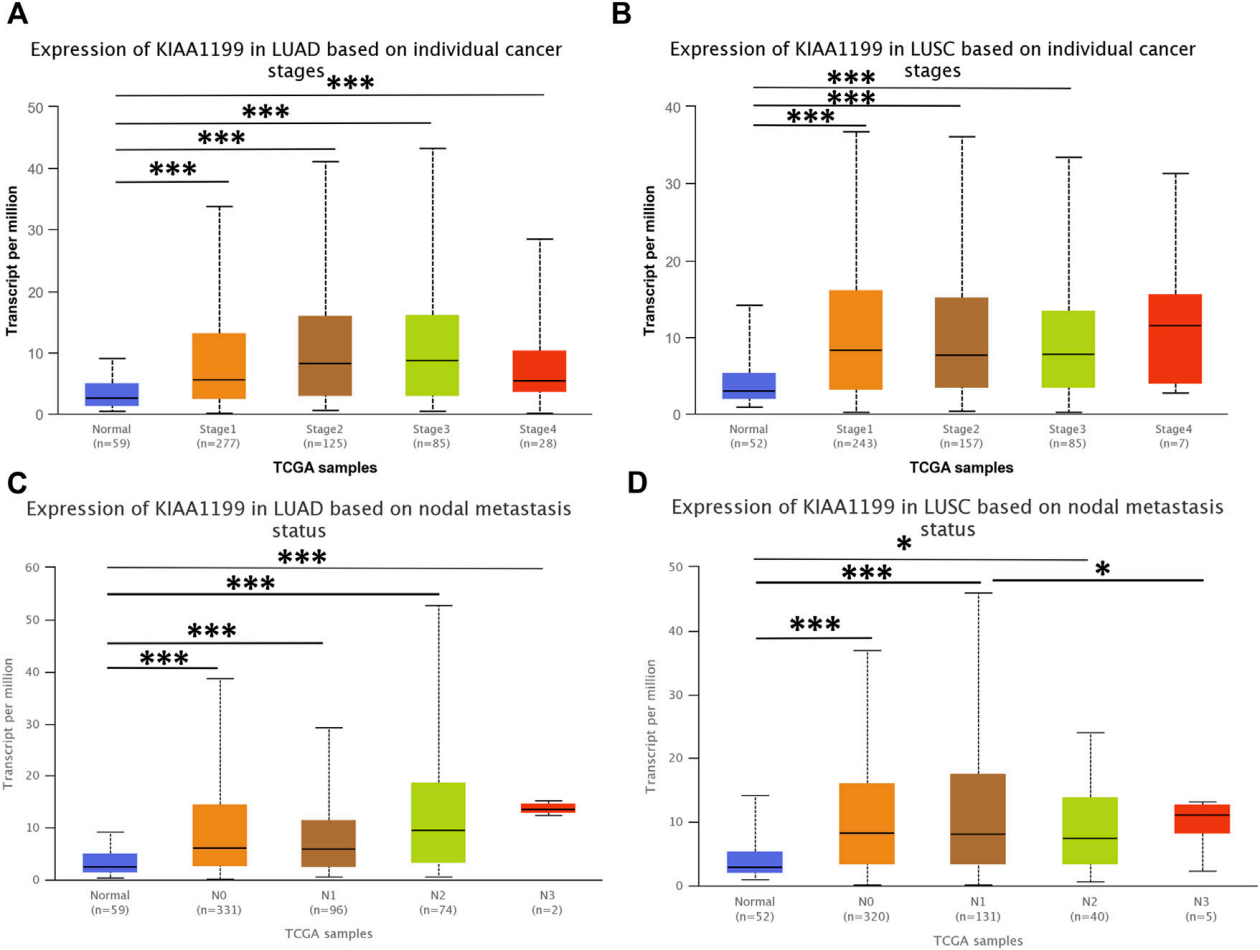
The correlations among the expression level of KIAA1199 and cancer stages and nodal metastasis status in LUAD and LUSC in UALCAN database. Expression levels of KIAA1199 in **(A)** LUAD and **(B)** LUSC based on different cancer stages. Expression levels of KIAA1199 in **(C)** LUAD and **(D)** LUSC based on individual cancer stages nodal metastasis status. *p < 0.05; **p < 0.01; ***p < 0.001.

The expression of KIAA1199 mRNA in pan-cancer was assessed by using TIMER and GEPIA databases. As shown in Figure 1A, based on TIMER database, the KIAA1199 mRNA expression was significantly up-regulated in 15 cancers compared with their matched normal tissues, such as breast invasive carcinoma (BRCA), colon adenocarcinoma (COAD), pancreatic adenocarcinoma (PAAD), stomach adenocarcinoma (STAD), and so on. The KIAA1199 mRNA expression was also significantly increased in LUAD and LUSC tissues, which was consistent with the results of GEPIA database (Figures 1B,C). Results from LCE database confirmed that KIAA1199 was overexpressed in LUAD and LUSC tissues (Figures 1D,E). Furthermore, we explored the relationships between expression of KIAA1199 and clinicopathological features of LUAD and LUSC samples based on UALCAN database. The expression level of KIAA1199 in LUAD and LUSC samples showed significant differences compared to normal tissues. However, there was no significant difference in the expression level of KIAA1199 between groups based on different stages (Figures 2A,B) and lymph node metastasis (Figures 2C,D)). The results proclaimed that KIAA1199 was overexpressed in LUAD and LUSC tissues, while the relationships between expression of KIAA1199 and clinicopathological features of LUAD and LUSC samples may need larger sample size to confirm.

### The Potential Effect of KIAA1199 on Survival Outcomes of LUAD and LUSC

**FIGURE 3:**
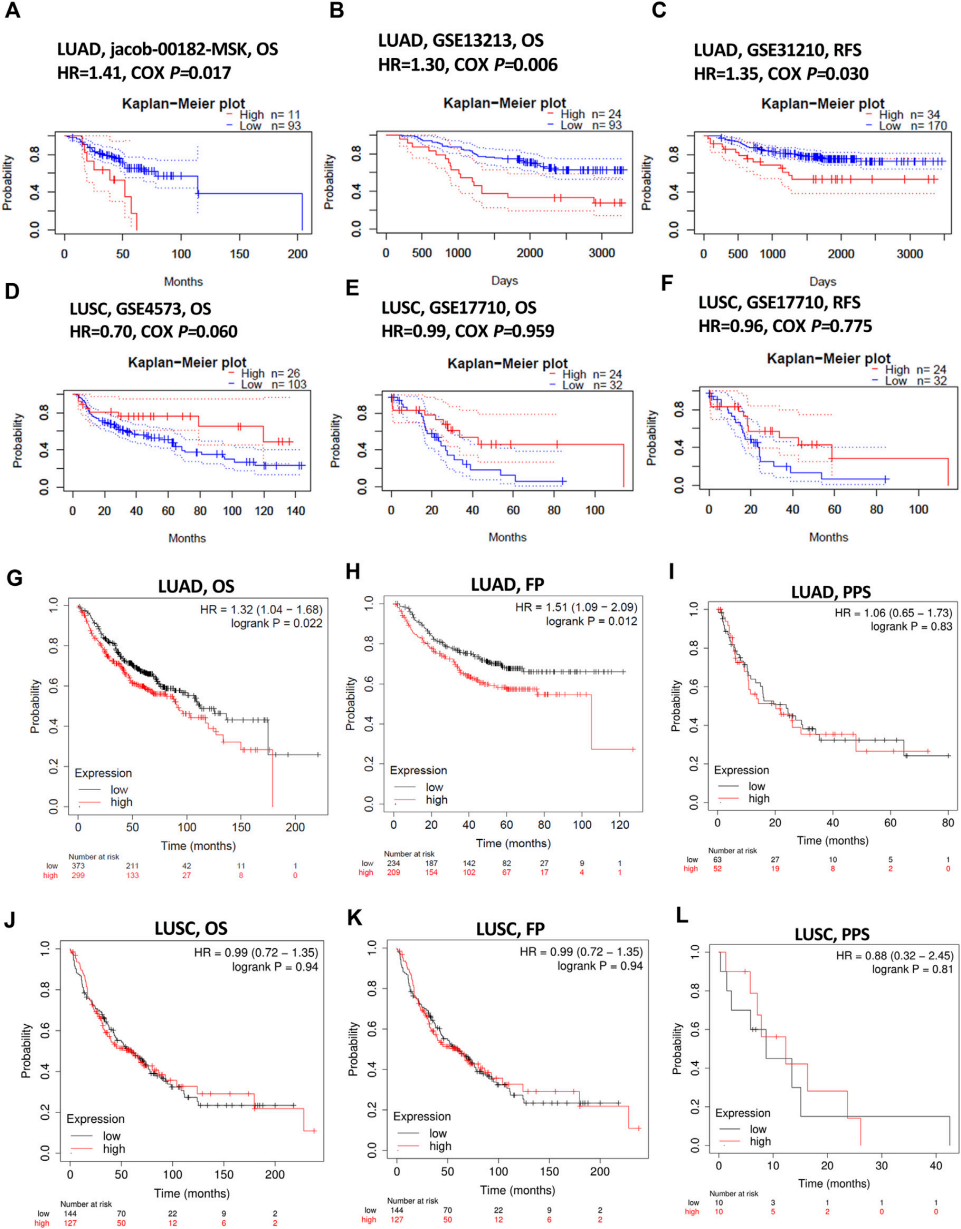
The prognostic role assessment of KIAA1199 for LUAD and LUSC based on Prognoscan and Kaplan-Meier plotter database. Survival curves of **(A,B)** OS (overall survival) in LUAD cohort (Jacob-00182-MSK, *n* = 104; GSE13213, *n* = 117). **(C)** RFS (relapse-free survival) in LUAD cohort (GSE31210, *n* = 204). **(D,E)** OS in LUSC cohort (GSE4573, *n* = 129; GSE17710, *n* = 56). **(F)** RFS in LUSC cohort (GSE17710, *n* = 56). **(G–I)** OS, FP (first progression) and PPS (post-progression survival) in LUAD patients (*n* = 672; *n* = 443; *n* = 115). **(J–L)** OS, FP and PPS in LUSC patients (*n* = 271; *n* = 271; *n* = 20).

Impact of KIAA1199 on lung cancer patient survival was investigated through the PrognoScan database. The patients were divided into two groups according to the expression level of KIAA1199: a low KIAA1199 group and a high KIAA1199 group. The results showed that high expression of KIAA1199 was correlated with relatively poor prognosis for overall survival (OS) and relapse-free survival (RFS) in LUAD (Jacob-00182-MSK: OS, HR = 1.41, *p* = 0.017; GSE13213: OS, HR = 1.30, *p* = 0.006; GSE31210: RFS, HR = 1.35, *p* = 0.03) (Figures 3A–C), while no significant correlation was displayed between patients’ OS, RFS and the expression level of KIAA1199 in LUSC (Figures 3D–F).

**FIGURE 4:**
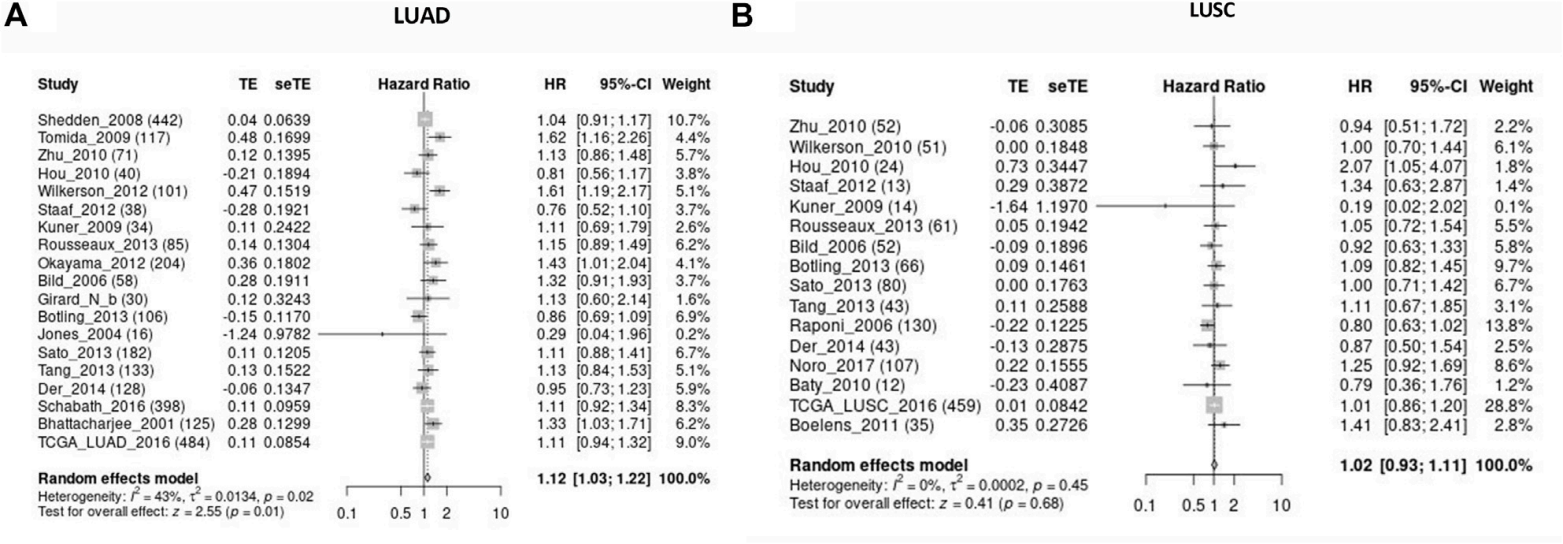
Validation for the prognostic role of KIAA1199 in LUAD and LUSC by the LCE database. The forest plot presented the results of survival meta-analyses of **(A)** LUAD patients. **(B)** LUSC patients. TE, estimate of treatment effect; seTE, standard error of treatment estimate; HR, hazard ratio; CI, confidence interval.

Kaplan-Meier plotter database was also used to evaluate the prognostic value of KIAA1199. The Kaplan-Meier (KM) survival curves demonstrated that higher expression of KIAA1199 was greatly associated with worse OS and first progression (FP) rate of LUAD patients (Figures 3G,H). While no significant correlation was observed among the expression level of KIAA1199 and LUSC patients’ OS, FP, Post-progression survival (PPS) (Figures 3J–L) as well as LUAD patients’ PPS (Figure 3I). Based on LCE database, the results of survival meta-analyses indicated the prognostic role of KIAA1199 in LUAD rather than LUSC (Figures 4A,B). These results showed that KIAA1199 might be accurate for determining the prognosis of LUAD patients.

### Experimental Verification of the KIAA1199 Expression Level in LUAD

**FIGURE 5:**
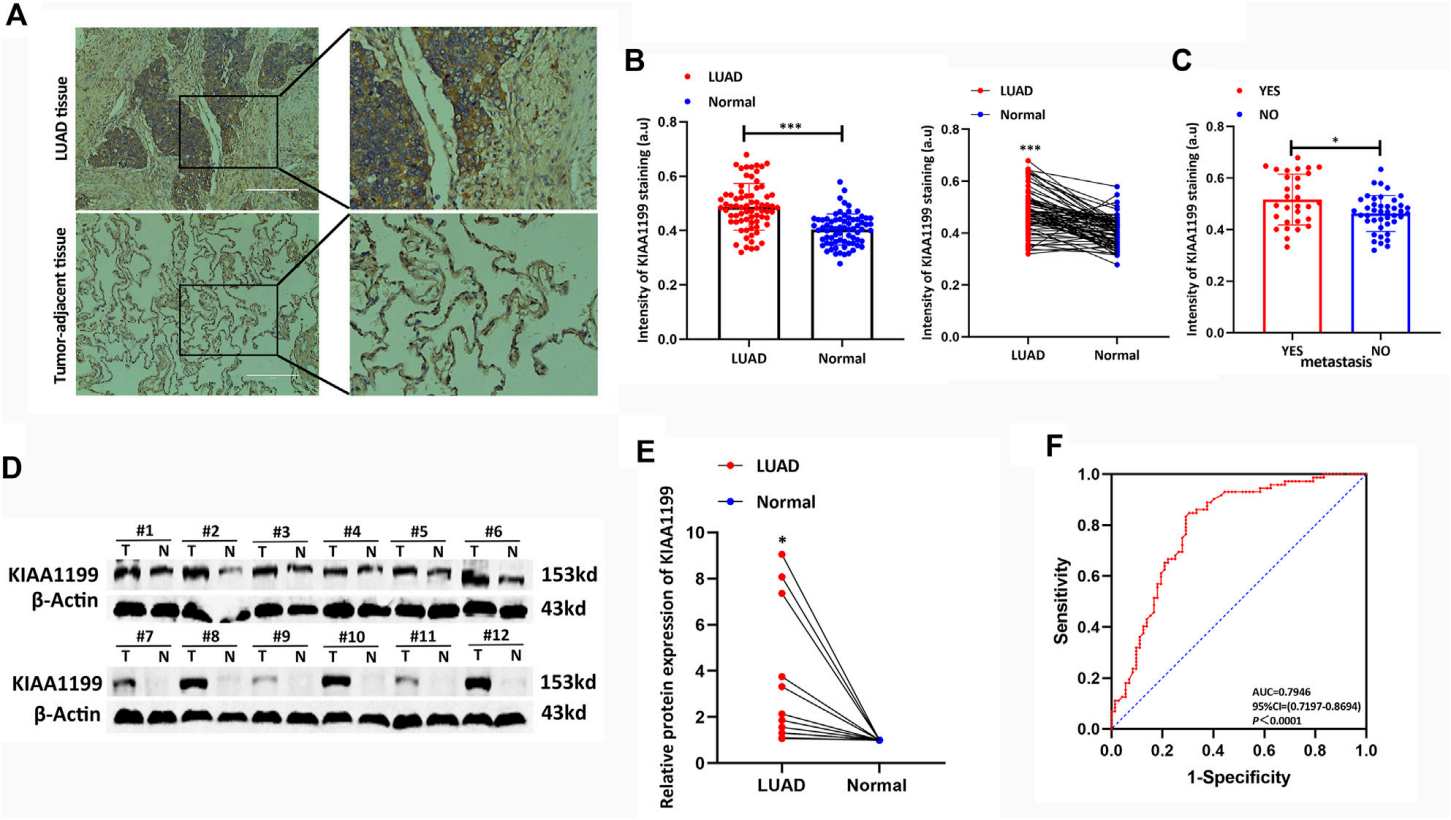
Experimental verification of the KIAA1199 expression in LUAD. **(A)** The representative immunohistochemical (IHC) images of KIAA1199 in LUAD clinical samples and their adjacent normal lung tissue (*n* = 72). **(B)** The staining intensity of KIAA1199. **(C)** The staining intensity of KIAA1199 for LUAD patients with or without metastasis. **(D,E)** The KIAA1199 protein expression level in 12 pairs of LUAD tumor tissues and adjacent normal lung tissues (T, tumor tissues; N, adjacent normal lung tissues) was displayed by bar chart. **(F)** Receiver operating characteristic (ROC) curve of KIAA1199 expression in 72 pairs of LUAD tumor tissues and adjacent normal lung tissues. Magnification ×200. *p < 0.05; **p < 0.01; ***p < 0.001.

In order to confirm the prognostic role of KIAA1199 in LUAD, 72 pairs of LUAD tumor tissues and para-cancerous non-tumor tissues and 12 pairs of fresh LUAD tissues were used for experimental validation at the protein level by IHC and WB, respectively. The results showed that KIAA1199 protein expression was significantly elevated in LUAD tissues compared with normal lung tissues (Figures 5A,B,D,E). In order to evaluate the association between clinicopathologic factors and patients’ outcomes, we conducted Univariate Kaplan-Meier survival analysis of OS for 72 patients with LUAD. As shown in Table 1, lymph node metastasis (31.07 ± 15.021 vs. 43.16 ± 15.539, *p* < 0.05) and high stage (31.00 ± 18.199 vs. 41.29 ± 13.805, *p* < 0.05) were associated with a significant poor OS as well as patients with KIAA1199 protein overexpression (36.48 ± 15.351 vs. 45.79 ± 15.982, *p* < 0.05). LUAD patients with metastasis also presented higher KIAA1199 expression than those without metastasis (Figure 5C). A multivariate Cox regression model analysis showed that lymph node metastasis was an independent factor of NSCLC prognosis (Supplementary Table S1). ROC curve analysis was conducted to evaluate the diagnostic value of KIAA1199, and the area under the curve (AUC) was 0.7710, which indicated that the high expression of KIAA1199 in LUAD showed preferable ability of discriminating LUAD from non-cancer lung samples (Figure 5F).

**TABLE 1:** Univariate Kaplan-Meier survival analysis of overall survival in 72 patients with LUAD.

	n	Univariate analysis of OS (M)		
		Mean ± SE (M)	95% CI	*P*
Gender				
Male	48	37.44 ± 15.645	32.89–41.98	0.520
Female	24	40 ± 16.312	33.11–46.89	
Age				
<60	33	36.03 ± 15.623	30.49–41.57	0.267
>60	39	40.21 ± 15.900	35.05–45.36	
KIAA1199 expression				
Low	14	45.79 ± 15.982	36.56–55.01	0.047*
High	58	36.48 ± 15.351	32.45–40.52	
T stage				
T1 T2	62	39.31 ± 14.592	35.60–43.01	0.330
T3 T4	10	32.00 ± 21.807	16.40–47.60	
N stage				
N = 0	43	43.16 ± 14.539	38.69–47.64	0.001**
N = 1 2 3	29	31.07 ± 15.021	25.36–36.78	
Stage I-IV				
I II	51	41.29 ± 13.805	37.41–45.18	0.011*
III IV	21	31.00 ± 18.199	22.72–39.28	

LUAD, lung adenocarcinoma; M, Months; OS, overall survival; CI, confidence interval; SE, standard error. *p < 0.05; **p < 0.01; ***p < 0.001.

### Genomic Mutation of KIAA1199 in LUAD

**FIGURE 6:**
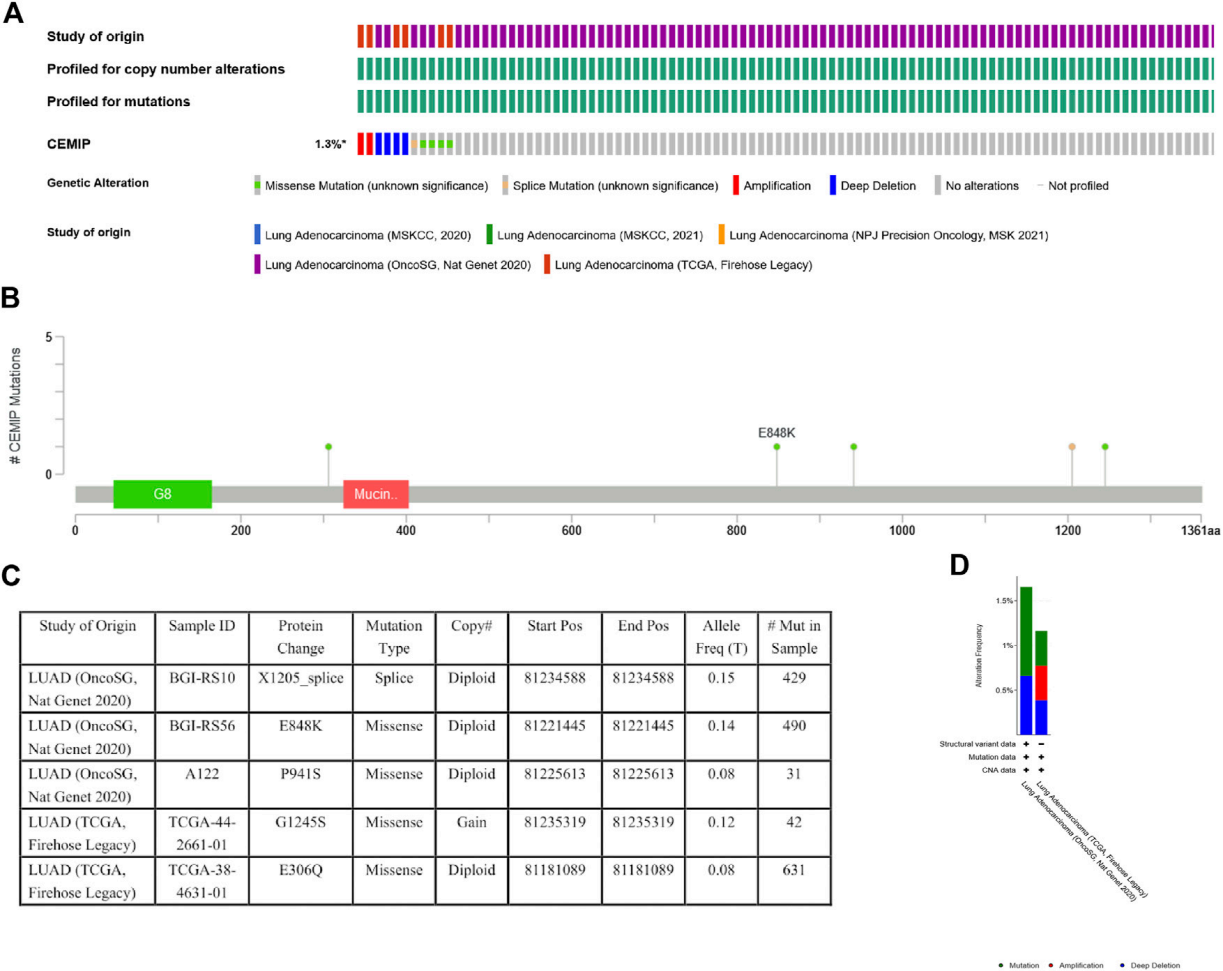
Mutation status view of KIAA1199 in LUAD. **(A)** OncoPrint of genomic alterations in KIAA1199 across a sample set. **(B,C)** Mutation sites of KIAA1199 in LUAD. **(D)** Genetic alteration frequency of KIAA1199 in LUAD studies.

Genetic variations of KIAA1199 in LUAD patients were analyzed based on the cBioPortal database. The ratio of KIAA1199 mutation, amplification, and deep deletion was 1.3%. Mutation was the main genetic alteration of KIAA1199 in LUAD (Figures 6A,D). In addition, the types and sites of KIAA1199 mutation in LUAD were further explored. As shown in Figures 6B,C, missense mutation of KIAA1199 resulted in the amino acid changes, because glutamic acid (E) 848 was replaced by lysine (K), proline (P) 941 was replaced by serine (S), glycine (G) 1245 was replaced by serine (S) or glutamic acid (E) 306 was replaced by glutamine (Q). Splice was found and contributed to protein change in LUAD, which presented as X1205_splice. These results indicated that genetic alterations of KIAA1199 could be found in LUAD.

### MiRNA and TFs Targets Related to KIAA1199 in LUAD

To further identify the molecular targets of KIAA1199 in LUAD, miRNAs and TFs enrichment of KIAA1199 were analyzed using the LinkedOmics database (Table 2). The top 5 most correlated miRNAs were (TTTGCAC) miR-19A, miR-19B (NES: 1.8495), (TTGCACT) miR-130A, miR-301, miR-130B (NES: 1.8407), (ACCAAAG) miR-9 (NES: 1.8224), (TAATAAT) miR-126 (NES: 1.7949), (CTCCAAG) miR-432 (NES: 1.7869). The top 5 most correlated TFs included V$SRF_C (NES: 1.98), V$RP58_01 (NES: 1.9686), V$SRF_Q6 (NES: 1.9369), V$SRF_Q5_01 (NES: 1.9309), V$FREAC4_01 (NES: 1.8938). These miRNAs and TFs might be involved in the regulation of transcription and expression of KIAA199.

**TABLE 2:** The miRNA- and transcription factor-target networks highly associated with KIAA1199 in LUAD.

Enriched category	Gene set	Size	Leading edge number	*p* value	FDR
miRNA Target	TTTGCAC, miR-19A, miR-19B	479	160	0****	0
	TTGCACT, miR-130A, miR-301, miR-130B	365	130	0****	0.001139
	ACCAAAG, miR-9	458	139	0****	0.000759
	TAATAAT, miR-126	207	71	0****	0.001898
	CTCCAAG, miR-432	75	32	0****	0.001952
Transcription Factor Target	V$SRF_C	201	84	0****	0
	V$RP58_01	194	78	0****	0
	V$SRF_Q6	231	81	0****	0
	V$SRF_Q5_01	207	78	0****	0
	V$FREAC4_01	140	45	0****	0

*P* or FDR, value equal to 0 means *p* < 0.000001. *p < 0.05; **p < 0.01; ***p < 0.001; ****p < 0.0001.

### Function and Pathway Enrichment Analysis of KIAA1199 in LUAD

**FIGURE 7:**
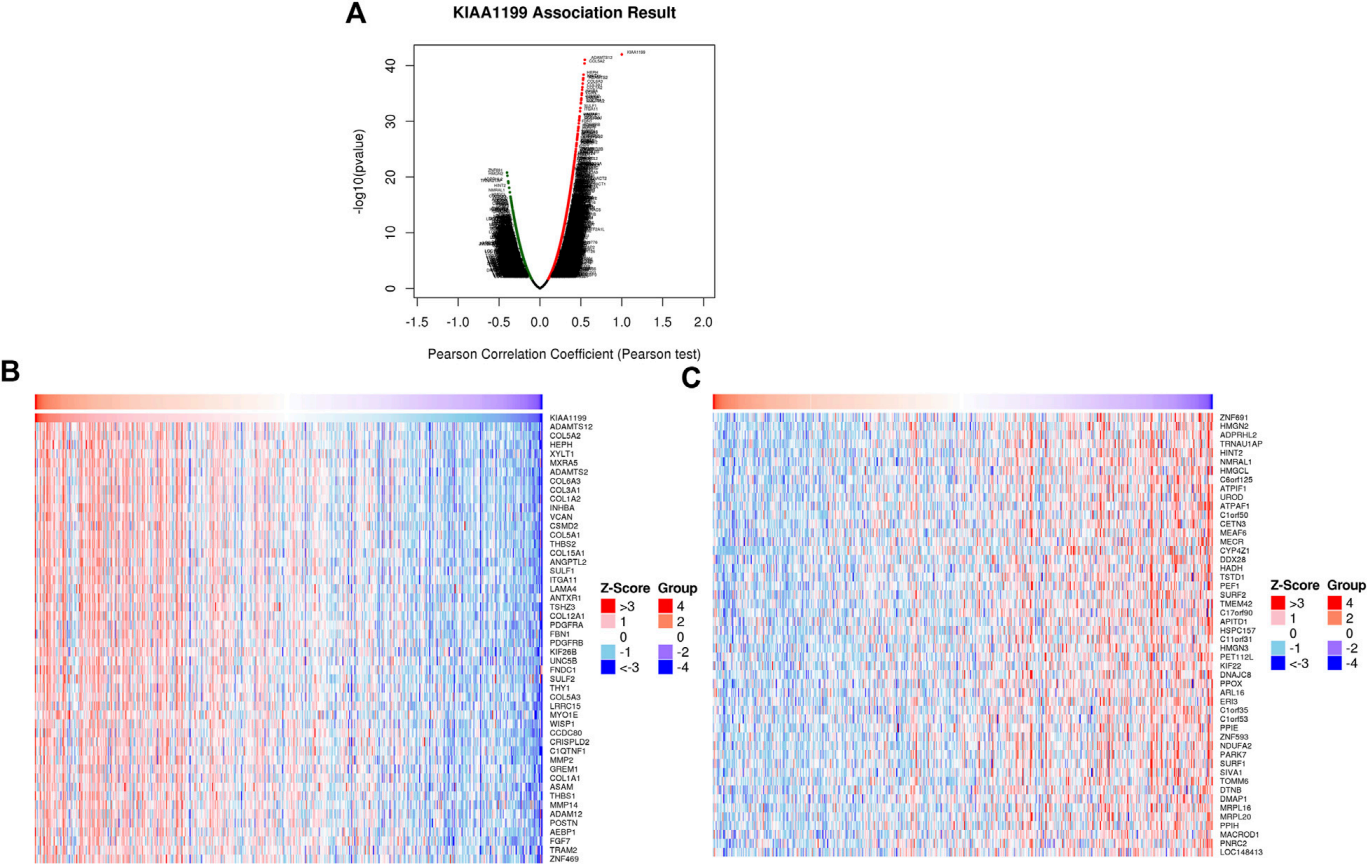
KIAA1199 co-expression genes in LUAD were identified using LinkedOmics database. **(A)** The Volcano Plot showed genes highly correlated with KIAA1199 in LUAD. Heat maps showed top 50 genes **(B)** positively and **(C)** negatively associated with KIAA1199 in LUAD, respectively.

In order to identify the potential biological roles and molecular mechanisms of KIAA1199 in LUAD, the gene co-expression network with KIAA1199 was investigated through the function module of LinkedOmics. Volcano plots showed that 3343 genes (dark red dots) were positively correlated with KIAA1199, while 2672 genes (dark green dots) were negatively correlated with KIAA1199 (Figure 7A). The heat map showed the top 50 significant genes that are positively and negatively related with KIAA1199 in LUAD, respectively. (Figures 7B,C).

**FIGURE 8:**
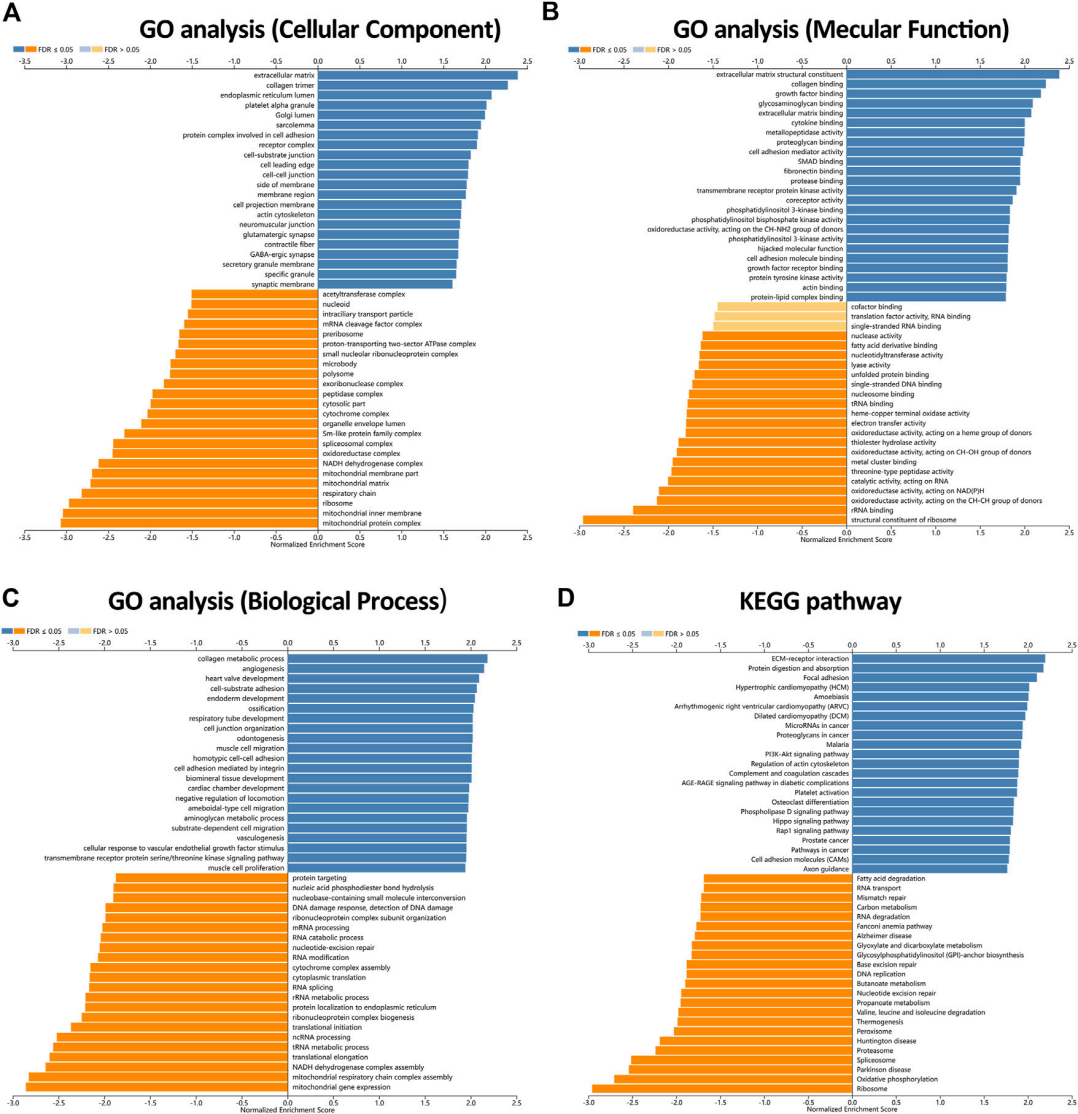
The enriched GO annotations and KEGG pathways analysis of KIAA1199 in LUAD from the LinkedOmics database. **(A)** GO biological process. **(B)** GO molecular function. **(C)** GO cellular components. **(D)** KEGG pathway. Dark blue and orange indicate FDR ≤0.05, light blue and orange indicate FDR >0.05.

To further uncover the potential role of KIAA1199 in LUAD, GO and KEGG analyses in TCGA dataset based on genes related to KIAA1199 were performed. The GO analysis showed that KIAA1199 co-expression genes were mainly localized in extracellular matrix and involved in extracellular matrix structure constituent collagen, growth factor and cytokine binding (Figures 8A,B). KIAA1199-related biological processes included collagen metabolic process, angiogenesis, cell-substrate adhesion and so on (Figure 8C). In addition, KEGG pathway annotation revealed that ECM-receptor interaction, focal adhesion, microRNAs in cancer, proteoglycans in cancer, PI3K-AKT signaling pathway were significant enriched pathways (Figure 8D).

### Associations Between KIAA1199 Expression and Immunological Status in LUAD Based on TIMER and TISIDB Databases

**FIGURE 9:**
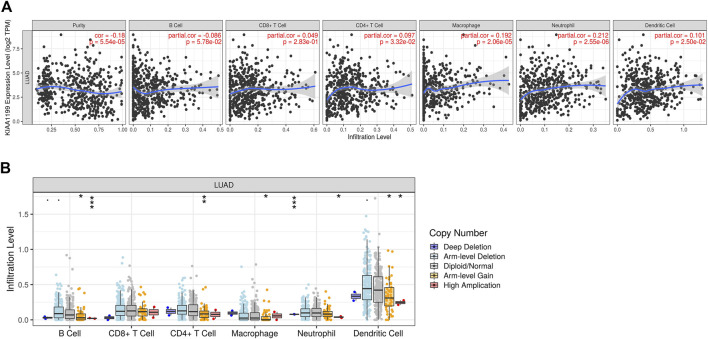
Association between KIAA1199 and the immune cells infiltration in LUAD based on the TIMER analysis. **(A)** The correlation between the KIAA1199 expression level and tumor purity, and the recruitments of macrophages, neutrophils, dendritic cells, CD4 + T cells, CD8 + T cells, and B cells in LUAD tissues. **(B)** The association of KIAA1199 copy number variation (CNV) with the B cells, CD8 + T cells, CD4 + T cells, macrophages, neutrophils, and dendritic cells in LUAD. *p < 0.05; **p < 0.01; ***p < 0.001.

The immune infiltration state in tumor microenvironment (TME) can affect patient prognosis, which is independent predictor for the outcome of immunotherapy and OS in LUAD patients. The relationship between KIAA1199 expression and the immune infiltration levels in LUAD was further evaluated using the TIMER database. The results indicated that KIAA1199 expression had a significant positive correlation with the infiltration levels of CD4^+^ T cells, macrophages, neutrophil cells, and dendritic cells in LUAD (Figure 9A), and the correlation with neutrophil cells was the strongest (cor = 0.212, *p* = 2.55e-06). KIAA1199 expression showed a significant positive relationship with CD11b (*p* = 4.11e-06), a gene marker of neutrophil cells (Supplementary Table S2). While the KIAA1199 level was significantly negatively correlated with tumor purity, which indicated that the tumor microenvironment may be a source of KIAA1199 expression. A comparison of the immune infiltration levels between LUAD with different somatic copy number variations (SCNVs) for KIAA1199 was performed. The SCNVs of KIAA1199 were significantly positively related to the infiltration of CD4^+^ T cells, B cells, neutrophils, macrophages, and dendritic cell (Figure 9B).

**FIGURE 10:**
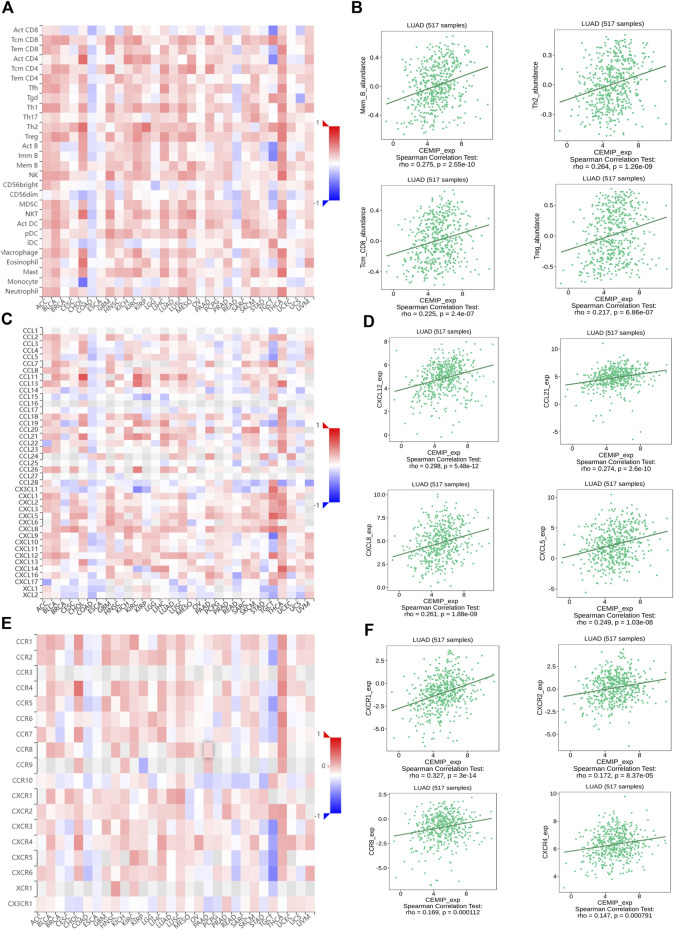
The correlations among KIAA1199 and tumor-infiltrating lymphocytes (TILs), chemokines and chemokine receptors in TISIDB database. Correlation of KIAA1199 expression with **(A,B)** 28 types of TILs; **(C,D)** chemokines; **(E,F)** chemokine receptors in LUAD.

Then, to validate the relationship between KIAA1199 expression and immune cell infiltration, we further investigated the correlations among KIAA1199 expression and TILs, multiple chemokines and chemokine receptors in LUAD patients using the TISIDB database. As shown in Figures 10A,B, KIAA1199 expression was positively correlated with the abundance of these types of TILs in LUAD, such as memory B cells (Mem B, rho = 0.275, *p* < 0.001), Central Memory CD8^+^ T cells (Tcm_CD8, rho = 0.225, *p* < 0.001), type 2 T helper cells (Th2, rho = 0.264, *p* < 0.001), Regulatory T cells (Treg, rho = 0.217, *p* < 0.001). The correlations among KIAA1199 expression and chemokines and their receptors were analyzed. A significant correlation was also observed among KIAA1199 and most of the chemokines and their receptors. The expression of KIAA1199 was positively correlated with the main chemokines such as CXCL12, CCL21, CXCL8, CXCL5 and the main chemokine receptors including CCR8, CCR4, CXCR1 and CXCR2 (Figures 10C–F). In summary, our results indicated that KIAA1199 might play a crucial role in tumor microenvironment immunoregulation in LUAD.

### Correlation Analysis Between Expression Level of KIAA1199 and Immune Cell Markers

Moreover, the correlation between KIAA1199 expression and the status of different immunosuppressive cells based on gene expression levels of immune cell markers was explored *via* the TIMER in LUAD. These immune marker genes included those for Th2, Treg, Tumor-associated macrophages (TAM), M2 macrophage, Myeloid-derived suppressor cell (MDSC), cancer-associated fibroblast (CAF), and T-cell exhaustion. The correlation adjustment in respect to purity or none was done in TIMER. Interestingly, KIAA1199 expression showed a positive correlation with expression of most markers of immunosuppressive cells, including TAM (CCL2, CD68 and IL10), M2 macrophage (CD163, VSIG4, and MS4A4A), Th2 (GATA3 and STAT5A), Treg (FOXP3, CCR8, STAT5B and TGFβ), T-cell exhaustion (CTLA-4 and TIM-3), MDSC (CD11b, CD39, CD31 and CD34), CAF (α-SMA, FAP, CD90 and MFAP5) (Table 3). In summary, the KIAA1199 expression was positively correlated with particular immunosuppressive cells. These results further indicated that KIAA1199 might participate in the recruitment of immunosuppressive cells to LUAD, leading to tumor progression and dismal patient prognosis.

**TABLE 3:** Correlation analysis between KIAA1199 and related genes of immunosuppressive cells markers in TIMER.

Description	Gene marker	LUAD			
		None		Purity	
		*P*		*P*	
TAM	CCL2	0.173	8.32E−05****	0.127	4.88E−03**
	CD68	0.229	1.67E−07****	0.186	3.13E−05****
	IL10	0.147	8.36E−04***	0.095	3.55E−02*
M2 macrophage	CD163	0.279	1.4E−10****	0.244	4.23E−08****
	VSIG4	0.132	2.75E−03**	0.09	4.52E−02*
	MS4A4A	0.189	1.71E−05****	0.141	1.74E−03**
Th2	GATA3	0.155	4.09E−04***	0.079	8.01E−02
	STAT6	0.063	1.55E−01	0.078	8.18E−02
	STAT5A	0.21	1.49E−06****	0.152	7.08E−04***
	IL13	−0.017	6.94E−01	−0.047	2.95E−01
Treg	FOXP3	0.207	2.23E−06****	0.138	2.13E−03**
	CCR8	0.252	7.09E−09****	0.198	9.73E−06****
	STAT5B	0.261	1.82E−09****	0.26	4.66E−09****
	TGFβ (TGFB1)	0.276	2.21E−10****	0.235	1.24E−07****
T-cell exhaustion	PD-1 (PDCD1)	0.071	1.06E−01	−0.011	0.801
	CTLA-4	0.107	1.53E−02*	0.022	6.33E−01
	LAG3	0.037	3.98E−01	−0.025	5.74E−01
	TIM-3 (HAVCR2)	0.161	2.38E−04***	0.092	4.09E−02*
MDSC	CD11b (ITGAM)	0.202	4.11E−06****	0.151	7.94E−04***
	CD39 (ENTPD1)	0.378	0E+00****	0.353	6.54E−16****
	CD31 (PECAM1)	0.274	2.87E−10****	0.228	3.29E−07****
	CD34	0.312	4.37E−13****	0.277	4.10E−10****
	CD33	0.078	7.52E−02	0.016	7.15E−01
CAF	α-SMA (ACTA2)	0.461	1.77E−28****	0.435	3.36E−24****
	FAP	0.423	8.34E−24****	0.392	1.32E−19****
	CD90 (THY1)	0.489	0E+00****	0.46	3.61E−27****
	MFAP5	0.366	9.18E−18****	0.345	3.40E−15****

*p< 0.05; **p < 0.01; ***p < 0.001; ****p < 0.0001.

## Discussion

Although some progresses have been made in exploring molecular targeted therapy and immunotherapy in lung cancer in recent years, the total survival time of patients is still less than 5 years due to high heterogeneity and lethality of lung cancer [5, 40]. KIAA1199 was initially identified as one of human novel large (>4 Kb) cDNAs in the Human Unidentified Gene-Encoded (HUGE) protein database [41]. Recently, several studies have indicated that KIAA1199 contributes to proliferation, adhesion, invasiveness and migration, drug resistance and poor prognosis of various carcinomas, including NSCLC [42–45]. However, a systematic analysis of KIAA1199 expression and its potential influence on clinical efficacy, biological significance and tumor immune microenvironment changes in NSCLC has not been performed yet. In this study, we performed an integrated analysis of KIAA1199 in NSCLC by using bioinformatics approaches and a variety of online analysis tools. Our results showed that KIAA1199 expression was significant higher in both LUAD and LUSC than that in normal lung tissues. Moreover, KIAA1199 may act as a promising biomarker for predicting prognosis in LUAD patients.

In our study, KIAA1199 gene alterations were found in LUAD, such as copy number amplification and missense mutation. Differentially mutated genes are considered as candidate cancer “driver” genes, and certain somatically mutated genes took part in tumor initiation and progression [46]. For example, EGFR activating mutations as in frame deletions in exon 19 or the Leu858Arg (L858R) point mutation in exon 21 are related with the therapeutic effect of first generation quinazoline reversible EGFR tyrosine kinase inhibitors (TKIs) in NSCLC patients. KIAA1199 mutation in the GG domain was reported to cause hearing loss due to the folding change of protein structure [13], meanwhile its carcinogenic effect was thought to be related to the overexpression of KIAA1199 [17, 20], suggesting that KIAA1199 gene copy number amplification is responsible for the malignant progression of LUAD. However, whether other gene alteration types, such as missense mutations, are involved in the malignant progression of LUAD is still unknown and needs to be further investigated.

MiRNAs are known to play important roles in many cellular biology activities such as cell death, regeneration, differentiation and gene expression. MiRNAs can act as tumor suppressor miRNAs to silence gene expression through binding to complementary sequences in the 3′ untranslated region (UTR) of their target mRNAs [47, 48], or act as oncomiRs to stimulate gene expression by specifically binding to 5 ′ -UTR of mRNA [49] in cancers. In most of the studies, miRNA usually functions as a tumor suppressor by reducing the protein expression of KIAA1199 to inhibit tumor progression [14, 50]. For example, miR-4677-3p directly bound to the 3′UTR region of KIAA1199 and inhibited its expression [14]. In our study, miR-19A, miR-19B, miR-130 family (miR-130b, miR-301a, and miR-301b) and miR-432 were positively correlated with the KIAA1199 expression level, which might be involved in oncogenic role of KIAA1199 in LUAD as oncomiRs to contribute to cancer progression [51–54]. Based on the LinkedOmics database analysis for KIAA1199-related TFs targets, strong association between the expression level of serum response factor (SRF) and KIAA1199 was found in LUAD. SRF has been shown to play a critical role in the promotion of oncogenesis and metastasis in various cancers [55–57], which indicate that SRF may participate in oncogenic role of KIAA1199 in the malignant progression of LUAD.

Cancer cells have a close interaction with the TME, which consists of ECM (comprising collagen, integrins, fibronectin and so on), stromal cells (such as CAFs), various infiltrating immune cells (comprising T and B lymphocytes, NK cells, and TAM and so on) and blood vessels [58]. Cancer cells can functionally shape their microenvironment through the secretion of various cytokines, chemokines, and other factors, leading to a reprogramming of the surrounding cells for example stromal cells or immune cells, enabling them to play a determinative role in tumor survival and progression [59]. In our study, the results of GO and KEGG pathway analyses showed that KIAA1199-related genes were predominantly enriched in the ECM-receptor interaction, and mostly involved in extracellular matrix structure constituent, collagen binding and metabolic process, growth factor and cytokine binding and so on. Consistently, KIAA1199 is a protein with hyaluronidase activity and is highly essential in the depolymerisation of hyaluronic acid (HA) to low-molecular-weight HA (LMW-HA) [12, 60]. As a primary component in ECM, HA or LMW-HA can be recognized by various types of cells including cancer cells through HA receptors (such as CD44, RHAMM, etc), thereby playing pivotal roles in many biological processes such as tumor invasion, proliferation, and inflammation [61]. Moreover, it has been reported that the expression of KIAA1199 was correlated with the production of type I collagen [62]. Type I collagen is frequently upregulated during tumorigenesis. By binding to its receptors on tumor cells, Type I collagen can promote tumor cell proliferation, epithelial-mesenchymal transition and metastasis, as well as regulate the efficacy of chemotherapy, radiotherapy and immunotherapy [63]. Therefore, consistent with these reports, our findings suggest that KIAA1199 may be involved in shaping a tumor-friendly TME, thereby accelerating cancer progression and deteriorating prognosis in LUAD patients.

In the present study, positive correlations among KIAA1199 expression and immune cell infiltration (particularly Mem B, Tcm_CD8, Th2, and Treg), immunosuppressive cell infiltration (TAMs, M2 macrophage, Th2, Treg, T-cell exhaustion, MDSCs and CAFs) were observed. Accumulation of evidence has documented that infiltration of these immunosuppressive cells in TME is associated with LUAD patients’ outcome. For instance, the infiltration of CAFs and M2 macrophages induces cancer progression [54, 64, 65]. Thus, our data suggest that as major producers of ECM and paracrine signals, CAFs are involved in the function of KIAA1199 in promoting cancer progression. However, whether CAFs-facilitated oncogenic role of KIAA1199 is related to ECM-receptor interaction or collagen binding and metabolic process still needs further exploration. It’s known that different immune cell subsets can be recruited into TME *via* interactions between chemokines and chemokine receptors, and affect tumor progression and therapeutic outcome [66]. Chemokines (CXCL12 [67], CXCL8 [68], CXCL5 [69]) and chemokine receptors (CXCR1/2 [70], CCR4 [71, 72], CCR8 [73]) are well-known to deeply participate in the tumor development and progression in various cancers, including LUAD [74]. In the present study, we found that the expression of KIAA1199 was correlated with the levels of many chemokines (CXCL12, CXCL8, CXCL5) and chemokine receptors (CXCR1/2, CXCR4, CCR8) in LUAD. Our results indicate that KIAA1199 may serve as a potential biomarker for evaluating the status of tumor immunity in LUAD.

## Conclusion

In conclusion, KIAA1199 has prognostic value in predicting unfavorable prognosis in LUAD. KIAA1199 has a close relationship with TME and might exert its oncogenic roles mainly through participating in the ECM formation, inducing immunosuppressive cell infiltration, as well as increasing chemokines and chemokine receptors levels. Thus, KIAA1199 may serve as a potential prognostic marker and a pivotal oncogene to promote tumor progression in LUAD.

## Data Availability

The original contributions presented in the study are included in the article/Supplementary Material, further inquiries can be directed to the corresponding authors.

## References

[1] Sung H Ferlay J Siegel RL Laversanne M Soerjomataram I Jemal A Global Cancer Statistics 2020: GLOBOCAN Estimates of Incidence and Mortality Worldwide for 36 Cancers in 185 Countries. CA Cancer J Clin (2021) 71(3):209–49. 10.3322/caac.21660 33538338

[2] Siegel RL Miller KD Fuchs HE Jemal A. Cancer Statistics. CA Cancer J Clin (2021) 71(1):7–33. 10.3322/caac.21654 33433946

[3] Herbst RS Morgensztern D and Boshoff C. The Biology and Management of Non-small Cell Lung Cancer. Nature (2018) 553(7689):446–54. 10.1038/nature25183 29364287

[4] Peinado-Serrano J, and Carnero A. Molecular Radiobiology in Non-small Cell Lung Cancer: Prognostic and Predictive Response Factors. Cancers (Basel) (2022) 14(9):2202. 10.3390/cancers14092202 35565331 PMC9101029

[5] Hirsch FR Scagliotti GV Mulshine JL Kwon R Curran WJ Jr Wu YL Lung Cancer: Current Therapies and New Targeted Treatments. Lancet (2017) 389(10066):299–311. 10.1016/s0140-6736(16)30958-8 27574741

[6] Wang M Herbst RS and Boshoff C. Toward Personalized Treatment Approaches for Non-small-cell Lung Cancer. Nat Med (2021) 27(8):1345–56. 10.1038/s41591-021-01450-2 34385702

[7] Doroshow DB Sanmamed MF Hastings K Politi K Rimm DL Chen L Immunotherapy in Non-small Cell Lung Cancer: Facts and Hopes. Clin Cancer Res (2019) 25(15):4592–602. 10.1158/1078-0432.ccr-18-1538 30824587 PMC6679805

[8] Schoenfeld AJ Antonia SJ Awad MM Felip E Gainor J Gettinger SN Clinical Definition of Acquired Resistance to Immunotherapy in Patients with Metastatic Non-small-cell Lung Cancer. Ann Oncol (2021) 32(12):1597–607. 10.1016/j.annonc.2021.08.2151 34487855 PMC12013006

[9] Lim Zf, and Ma PC. Emerging Insights of Tumor Heterogeneity and Drug Resistance Mechanisms in Lung Cancer Targeted Therapy. J Hematol Oncol (2019) 12(1):134. 10.1186/s13045-019-0818-2 31815659 PMC6902404

[10] Xu JY Zhang C Wang X Zhai L Ma Y Mao Y Integrative Proteomic Characterization of Human Lung Adenocarcinoma. Cell (2020) 182(1):245–61. 10.1016/j.cell.2020.05.043 32649877

[11] Terlizzi M Colarusso C Pinto A and Sorrentino R. Drug Resistance in Non-small Cell Lung Cancer (NSCLC): Impact of Genetic and Non-genetic Alterations on Therapeutic Regimen and Responsiveness. Pharmacol Ther (2019) 202:140–8. 10.1016/j.pharmthera.2019.06.005 31226345

[12] Zhang W Yin G Zhao H Ling H Xie Z Xiao C Secreted KIAA1199 Promotes the Progression of Rheumatoid Arthritis by Mediating Hyaluronic Acid Degradation in an ANXA1-dependent Manner. Cell Death Dis (2021) 12(1):102. 10.1038/s41419-021-03393-5 33473125 PMC7817834

[13] Abe S Usami S and Nakamura Y. Mutations in the Gene Encoding KIAA1199 Protein, an Inner-Ear Protein Expressed in Deiters' Cells and the Fibrocytes, as the Cause of Nonsyndromic Hearing Loss. J Hum Genet (2003) 48(11):564–70. 10.1007/s10038-003-0079-2 14577002

[14] Mi C Zhang D Li Y Ren M Ma W Lu G miR-4677-3p Participates Proliferation and Metastases of Gastric Cancer Cell via CEMIP-Pi3k/AKT Signaling Pathway. Cell Cycle (2021) 20(19):1978–87. 10.1080/15384101.2021.1971375 34437815 PMC8565831

[15] Xie G Dong P Chen H Xu L Liu Y Ma Y Decreased Expression of ATF3, Orchestrated by β-catenin/TCF3, miR-17-5p and HOXA11-AS, Promoted Gastric Cancer Progression via Increased β-catenin and CEMIP. Exp Mol Med (2021) 53(11):1706–22. 10.1038/s12276-021-00694-9 34728784 PMC8639750

[16] Song M Liu J Zheng X Zhou X Feng Z and Hu W. MiR-148a-3p Targets CEMIP to Suppress the Genesis of Gastric Cancer Cells. Biochem Biophys Res Commun (2021) 575:42–9. 10.1016/j.bbrc.2021.08.039 34455220

[17] Oba T Sato N Adachi Y Amaike T Kudo Y Koga A Hypoxia Increases KIAA1199/CEMIP Expression and Enhances Cell Migration in Pancreatic Cancer. Sci Rep (2021) 11(1):18193. 10.1038/s41598-021-97752-z 34521918 PMC8440617

[18] Zhang D Zhao L Shen Q Lv Q Jin M Ma H Down-regulation of KIAA1199/CEMIP by miR-216a Suppresses Tumor Invasion and Metastasis in Colorectal Cancer. Int J Cancer (2017) 140(10):2298–309. 10.1002/ijc.30656 28213952

[19] Hua Q Zhang B Xu G Wang L Wang H Lin Z CEMIP, a Novel Adaptor Protein of OGT, Promotes Colorectal Cancer Metastasis through Glutamine Metabolic Reprogramming via Reciprocal Regulation of β-catenin. Oncogene (2021) 40(46):6443–55. 10.1038/s41388-021-02023-w 34608265

[20] Zhang P Song Y Sun Y Li X Chen L Yang L AMPK/GSK3β/β-catenin cascade-triggered Overexpression of CEMIP Promotes Migration and Invasion in Anoikis-Resistant Prostate Cancer Cells by Enhancing Metabolic Reprogramming. Faseb j (2018) 32(7):3924–35. 10.1096/fj.201701078R 29505302

[21] Yu Y Liu B Li X Lu D Yang L Chen L ATF4/CEMIP/PKCα Promotes Anoikis Resistance by Enhancing Protective Autophagy in Prostate Cancer Cells. Cel Death Dis (2022) 13(1):46. 10.1038/s41419-021-04494-x PMC8748688 35013120

[22] Xu Y Xu H Li M Wu H Guo Y Chen J KIAA1199 Promotes Sorafenib Tolerance and the Metastasis of Hepatocellular Carcinoma by Activating the EGF/EGFR-dependent Epithelial-Mesenchymal Transition Program. Cancer Lett (2019) 454:78–89. 10.1016/j.canlet.2019.03.049 30980868

[23] Liu B Li X Wang D Yu Y Lu D Chen L CEMIP Promotes Extracellular Matrix-Detached Prostate Cancer Cell Survival by Inhibiting Ferroptosis. Cancer Sci (2022) 113(6):2056–70. 10.1111/cas.15356 35363929 PMC9207355

[24] Rodrigues G Hoshino A Kenific CM Matei IR Steiner L Freitas D Tumour Exosomal CEMIP Protein Promotes Cancer Cell Colonization in Brain Metastasis. Nat Cel Biol (2019) 21(11):1403–12. 10.1038/s41556-019-0404-4 PMC7354005 31685984

[25] Wang H Zhang B Li R Chen J Xu G Zhu Y KIAA1199 Drives Immune Suppression to Promote Colorectal Cancer Liver Metastasis by Modulating Neutrophil Infiltration. Hepatology (2022) 76:967–81. 10.1002/hep.32383 35108400

[26] Tang Z Ding Y Shen Q Zhang C Li J Nazar M KIAA1199 Promotes Invasion and Migration in Non-small-cell Lung Cancer (NSCLC) via PI3K-Akt Mediated EMT. J Mol Med (Berl) (2019) 97(1):127–40. 10.1007/s00109-018-1721-y 30478628

[27] Wang A Zhu J Li J Du W Zhang Y Cai T Downregulation of KIAA1199 by miR-486-5p Suppresses Tumorigenesis in Lung Cancer. Cancer Med (2020) 9(15):5570–86. 10.1002/cam4.3210 32519472 PMC7402811

[28] Li T Fan J Wang B Traugh N Chen Q Liu JS TIMER: A Web Server for Comprehensive Analysis of Tumor-Infiltrating Immune Cells. Cancer Res (2017) 77(21):e108–10. 10.1158/0008-5472.CAN-17-0307 29092952 PMC6042652

[29] Li C Tang Z Zhang W Ye Z and Liu F. GEPIA2021: Integrating Multiple Deconvolution-Based Analysis into GEPIA. Nucleic Acids Res (2021) 49(W1):W242–w246. 10.1093/nar/gkab418 34050758 PMC8262695

[30] Chand rashekar DS Bashel B Balasubramanya SAH Creighton CJ Ponce-Rodriguez I Chakravarthi B UALCAN: A Portal for Facilitating Tumor Subgroup Gene Expression and Survival Analyses. Neoplasia (2017) 19(8):649–58. 10.1016/j.neo.2017.05.002 28732212 PMC5516091

[31] Lánczky A, and Győrffy B Gyorffy B. Web-Based Survival Analysis Tool Tailored for Medical Research (KMplot): Development and Implementation. J Med Internet Res (2021) 23(7):e27633. 10.2196/27633 34309564 PMC8367126

[32] Mizuno H Kitada K Nakai K and Sarai A. PrognoScan: a New Database for Meta-Analysis of the Prognostic Value of Genes. BMC Med Genomics (2009) 2:18. 10.1186/1755-8794-2-18 19393097 PMC2689870

[33] Cai L Lin S Girard L Zhou Y Yang L Ci B LCE: an Open Web portal to Explore Gene Expression and Clinical Associations in Lung Cancer. Oncogene (2019) 38(14):2551–64. 10.1038/s41388-018-0588-2 30532070 PMC6477796

[34] Gao J Aksoy BA Dogrusoz U Dresdner G Gross B Sumer SO Integrative Analysis of Complex Cancer Genomics and Clinical Profiles Using the cBioPortal. Sci Signal (2013) 6(269):pl1. 10.1126/scisignal.2004088 23550210 PMC4160307

[35] Vasaikar SV Straub P Wang J and Zhang B. LinkedOmics: Analyzing Multi-Omics Data within and across 32 Cancer Types. Nucleic Acids Res (2018) 46(D1):D956-D963–d963. 10.1093/nar/gkx1090 29136207 PMC5753188

[36] Ru B Wong CN Tong Y Zhong JY Zhong SSW Wu WC TISIDB: an Integrated Repository portal for Tumor-Immune System Interactions. Bioinformatics (2019) 35(20):4200–2. 10.1093/bioinformatics/btz210 30903160

[37] Wei T Song J Liang K Li L Mo X Huang Z Identification of a Novel Therapeutic Cand idate, NRK, in Primary Cancer-Associated Fibroblasts of Lung Adenocarcinoma Microenvironment. J Cancer Res Clin Oncol (2021) 147(4):1049–64. 10.1007/s00432-020-03489-z 33387038 PMC11802160

[38] Li L Pan Y Mo X Wei T Song J Luo M A Novel Metastatic Promoter CEMIP and its Downstream Molecular Targets and Signaling Pathway of Cellular Migration and Invasion in SCLC Cells Based on Proteome Analysis. J Cancer Res Clin Oncol (2020) 146(10):2519–34. 10.1007/s00432-020-03308-5 32648226 PMC11804513

[39] Liu M Zhao SQ Yang L Li X Song X Zheng Y A Direct Immunohistochemistry (IHC) Method Improves the Intraoperative Diagnosis of Breast Papillary Lesions Including Breast Cancer. Discov Med (2019) 28(151):29–37. 31465723

[40] de Sousa Vml, and Carvalho L. Heterogeneity in Lung Cancer. Pathobiology (2018) 85(1-2):96–107. 10.1159/000487440 29635240

[41] Kikuno R Nagase T Nakayama M Koga H Okazaki N Nakajima D HUGE: a Database for Human KIAA Proteins, a 2004 Update Integrating HUGEppi and ROUGE. Nucleic Acids Res (2004) 32, D502–4. 10.1093/nar/gkh035 14681467 PMC308769

[42] Liu J Yan W Han P and Tian D. The Emerging Role of KIAA1199 in Cancer Development and Therapy. Biomed Pharmacother (2021) 138:111507. 10.1016/j.biopha.2021.111507 33773462

[43] Chen Y Zhou H Jiang WJ Wang JF Tian Y Jiang Y The Role of CEMIP in Tumors: An Update Based on Cellular and Molecular Insights. Biomed Pharmacother (2022) 146:112504. 10.1016/j.biopha.2021.112504 34922110

[44] Cheng J Zhang Y Wan R Zhou J Wu X Fan Q CEMIP Promotes Osteosarcoma Progression and Metastasis Through Activating Notch Signaling Pathway. Front Oncol (2022) 12:919108. 10.3389/fonc.2022.919108 35957875 PMC9361750

[45] Hsieh IY He J Wang L Lin B Liang Z Lu B H3K27me3 Loss Plays a Vital Role in CEMIP Mediated Carcinogenesis and Progression of Breast Cancer with Poor Prognosis. Biomed Pharmacother (2020) 123:109728. 10.1016/j.biopha.2019.109728 31846842

[46] Przytycki Pf, and Singh M. Differential Analysis between Somatic Mutation and Germline Variation Profiles Reveals Cancer-Related Genes. Genome Med (2017) 9(1):79. 10.1186/s13073-017-0465-6 28841835 PMC5574113

[47] Hayes J Peruzzi PP and Lawler S. MicroRNAs in Cancer: Biomarkers, Functions and Therapy. Trends Mol Med (2014) 20(8):460–9. 10.1016/j.molmed.2014.06.005 25027972

[48] Lee Y Dutta A. MicroRNAs in Cancer. Annu Rev Pathol (2009) 4:199–227. 10.1146/annurev.pathol.4.110807.092222 18817506 PMC2769253

[49] O'Brien J Hayder H Zayed Y and Peng C. Overview of MicroRNA Biogenesis, Mechanisms of Actions, and Circulation. Front Endocrinol (Lausanne) (2018) 9:402. 10.3389/fendo.2018.00402 30123182 PMC6085463

[50] Wang L Yu T Li W Li M Zuo Q Zou Q The miR-29c-Kiaa1199 axis Regulates Gastric Cancer Migration by Binding with WBP11 and PTP4A3. Oncogene (2019) 38(17):3134–50. 10.1038/s41388-018-0642-0 30626935

[51] Monoe Y Jingushi K Kawase A Hirono T Hirose R Nakatsuji Y Pharmacological Inhibition of miR-130 Family Suppresses Bladder Tumor Growth by Targeting Various Oncogenic Pathways via PTPN1. Int J Mol Sci (2021) 22(9):4751. 10.3390/ijms22094751 33947152 PMC8124864

[52] Li S Jia H Zhang Z and Wu D. DRAIC Promotes Growth of Breast Cancer by Sponging miR-432-5p to Upregulate SLBP. Cancer Gene Ther (2021) 29:951–60. 10.1038/s41417-021-00388-4 34645975

[53] Liu Y Liu R Yang F Cheng R Chen X Cui S miR-19a Promotes Colorectal Cancer Proliferation and Migration by Targeting TIA1. Mol Cancer (2017) 16(1):53. 10.1186/s12943-017-0625-8 28257633 PMC5336638

[54] Chen J Zhang K Zhi Y Wu Y Chen B Bai J Tumor-derived Exosomal miR-19b-3p Facilitates M2 Macrophage Polarization and Exosomal LINC00273 Secretion to Promote Lung Adenocarcinoma Metastasis via Hippo Pathway. Clin Transl Med (2021) 11(9):e478. 10.1002/ctm2.478 34586722 PMC8435259

[55] Müller S Glaß M Singh AK Haase J Bley N Fuchs T IGF2BP1 Promotes SRF-dependent Transcription in Cancer in a m6A- and miRNA-dependent Manner. Nucleic Acids Res (2019) 47(1):375–90. 10.1093/nar/gky1012 30371874 PMC6326824

[56] Li T Wan Y Su Z Li J Han M and Zhou C. SRF Potentiates Colon Cancer Metastasis and Progression in a microRNA-214/ptk6-dependent Manner. Cancer Manag Res (2020) 12:6477–91. 10.2147/cmar.s257422 32801887 PMC7395694

[57] Wang HY Zhang B Zhou JN Wang DX Xu YC Zeng Q Arsenic Trioxide Inhibits Liver Cancer Stem Cells and Metastasis by Targeting SRF/MCM7 Complex. Cel Death Dis (2019) 10(6):453. 10.1038/s41419-019-1676-0 PMC6560089 31186405

[58] Anderson Nm, and Simon MC. The Tumor Microenvironment. Curr Biol (2020) 30(16):R921-R925–r925. 10.1016/j.cub.2020.06.081 32810447 PMC8194051

[59] Hinshaw Dc, and Shevde LA. The Tumor Microenvironment Innately Modulates Cancer Progression. Cancer Res (2019) 79(18):4557–66. 10.1158/0008-5472.can-18-3962 31350295 PMC6744958

[60] Yoshida H Nagaoka A Kusaka-Kikushima A Tobiishi M Kawabata K Sayo T KIAA1199, a Deafness Gene of Unknown Function, Is a New Hyaluronan Binding Protein Involved in Hyaluronan Depolymerization. Proc Natl Acad Sci U S A (2013) 110(14):5612–7. 10.1073/pnas.1215432110 23509262 PMC3619336

[61] Huang J Zhang L Wan D Zhou L Zheng S Lin S Extracellular Matrix and its Therapeutic Potential for Cancer Treatment. Signal Transduct Target Ther (2021) 6(1):153. 10.1038/s41392-021-00544-0 33888679 PMC8062524

[62] Kwapiszewska G Gungl A Wilhelm J Marsh LM Thekkekara Puthenparampil H Sinn K Transcriptome Profiling Reveals the Complexity of Pirfenidone Effects in Idiopathic Pulmonary Fibrosis. Eur Respir J (2018) 52(5):1800564. 10.1183/13993003.00564-2018 30166321

[63] Shi R Zhang Z Zhu A Xiong X Zhang J Xu J Targeting Type I Collagen for Cancer Treatment. Int J Cancer (2022) 151(5):665–83. 10.1002/ijc.33985 35225360

[64] Xiang H Ramil CP Hai J Zhang C Wang H Watkins AA Cancer-Associated Fibroblasts Promote Immunosuppression by Inducing ROS-Generating Monocytic MDSCs in Lung Squamous Cell Carcinoma. Cancer Immunol Res (2020) 8(4):436–50. 10.1158/2326-6066.cir-19-0507 32075803

[65] Bremnes RM Dønnem T Al-Saad S Al-Shibli K Andersen S Sirera R The Role of Tumor Stroma in Cancer Progression and Prognosis: Emphasis on Carcinoma-Associated Fibroblasts and Non-small Cell Lung Cancer. J Thorac Oncol (2011) 6(1):209–17. 10.1097/JTO.0b013e3181f8a1bd 21107292

[66] Nagarsheth N Wicha MS and Zou W. Chemokines in the Cancer Microenvironment and Their Relevance in Cancer Immunotherapy. Nat Rev Immunol (2017) 17(9):559–72. 10.1038/nri.2017.49 28555670 PMC5731833

[67] Wu J Liu X Wu J Lou C Zhang Q Chen H CXCL12 Derived from CD248-Expressing Cancer-Associated Fibroblasts Mediates M2-Polarized Macrophages to Promote Nonsmall Cell Lung Cancer Progression. Biochim Biophys Acta Mol Basis Dis (2022) 1868(11):166521. 10.1016/j.bbadis.2022.166521 35985448

[68] Xue J Song Y Xu W and Zhu Y. The CDK1-Related lncRNA and CXCL8 Mediated Immune Resistance in Lung Adenocarcinoma. Cells (2022) 11(17):2688. 10.3390/cells11172688 36078096 PMC9454767

[69] Lu Q Yin H Deng Y Chen W Diao W Ding M circDHTKD1 Promotes Lymphatic Metastasis of Bladder Cancer by Upregulating CXCL5. Cell Death Discov (2022) 8(1):243. 10.1038/s41420-022-01037-x 35504887 PMC9065127

[70] Santolla MF Talia M Cirillo F Scordamaglia D De Rosis S Spinelli A The AGEs/RAGE Transduction Signaling Prompts IL-8/CXCR1/2-Mediated Interaction between Cancer-Associated Fibroblasts (CAFs) and Breast Cancer Cells. Cells (2022) 11(15):2402. 10.3390/cells11152402 35954247 PMC9368521

[71] Baer C Kimura S Rana MS Kleist AB Flerlage T Feith DJ CCL22 Mutations Drive Natural Killer Cell Lymphoproliferative Disease by Deregulating Microenvironmental Crosstalk. Nat Genet (2022) 54(5):637–48. 10.1038/s41588-022-01059-2 35513723 PMC9117519

[72] Sarkar T Dhar S Chakraborty D Pati S Bose S Pand a AK FOXP3/HAT1 Axis Controls Treg Infiltration in the Tumor Microenvironment by Inducing CCR4 Expression in Breast Cancer. Front Immunol (2022) 13:740588. 10.3389/fimmu.2022.740588 35222362 PMC8863663

[73] Wang T Zhou Q Zeng H Zhang H Liu Z Shao J CCR8 Blockade Primes Anti-tumor Immunity through Intratumoral Regulatory T Cells Destabilization in Muscle-Invasive Bladder Cancer. Cancer Immunol Immunother (2020) 69(9):1855–67. 10.1007/s00262-020-02583-y 32367308 PMC11027714

[74] Xu J Li JQ Chen QL Shestakova EA Misyurin VA Pokrovsky VS Advances in Research on the Effects and Mechanisms of Chemokines and Their Receptors in Cancer. Front Pharmacol (2022) 13:920779. 10.3389/fphar.2022.920779 35770088 PMC9235028

