# Supplementary material for: KIAA1199 Correlates With Tumor Microenvironment and Immune Infiltration in Lung Adenocarcinoma as a Potential Prognostic Biomarker
Source: Pathol Oncol Res. 2022 Nov 7;28:1610754. doi: 10.3389/pore.2022.1610754 (PMC9676226; doi:10.3389/pore.2022.1610754)
Supplement: Supplementary file 1 [file Table1.docx]

Table S1. Multivariate Cox regression model of overall

survival in 72 patients with LUAD

|  | n | Multivariate Cox analysis of OS (M) | |
| --- | --- | --- | --- |
|  |  | RR (95% CI) | *P* |
| KIAA1199 expression |  |  |  |
| Low | 14 | 1 |  |
| High | 58 | 2.679 (0.753-9.526) | 0.128 |
| N stage |  |  |  |
| N=0 | 43 | 1 |  |
| N=1 2 3 | 29 | 3.547 (1.318-9.543) | 0.012* |
| Stage I-IV |  |  |  |
| I II | 51 | 1 | 0.113 |
| III IV | 21 | 2.321 (0.819-6.584) |  |

M=Months, OS=Overall survival, RR=Relative risk, CI=Confidence interval. **p*<0.05; ** *p* <0.01; *** *p* <0.001.
