# Supplementary material for: KIAA1199 Correlates With Tumor Microenvironment and Immune Infiltration in Lung Adenocarcinoma as a Potential Prognostic Biomarker
Source: Pathol Oncol Res. 2022 Nov 7;28:1610754. doi: 10.3389/pore.2022.1610754 (PMC9676226; doi:10.3389/pore.2022.1610754)
Supplement: Supplementary file 2 [file Table2.docx]

Table S2. The relationship between biomarker genes of immune cell and KIAA1199 based on TIMER

| **Description** | **Gene marker** | | LUAD | | | | |
| --- | --- | --- | --- | --- | --- | --- | --- |
|  |  |  | **None** | |  | **Purity** | |
|  |  |  | ***P*** | |  | ***P*** | |
| CD8+ T cell | CD8A | | 0.045 | 3.09E-01 |  | -0.037 | 4.06E-01 |
|  | CD8B | | -0.007 | 8.70E-01 |  | -0.072 | 1/10E-01 |
| T cell (general) | CD3D | | 0.035 | 4.23E-01 |  | -0.072 | 1.10E-01 |
|  | CD3E | | 0.07 | 1.15E-01 |  | -0.038 | 4.06E-01 |
|  | | CD2 | 0.029 | 5.15E-01 |  | -0.082 | 6.79E-02 |
| B cell | CD19 | | 0.097 | 2.74E-02* |  | 0.012 | 7.89E-01 |
|  | CD79A | | 0.186 | 2.24E-05**** |  | 0.123 | 6.39E-03** |
| Monocyte | CD86 | | 0.209 | 1.80E-06**** |  | 0.15 | 8.14E-04*** |
|  | CD115 (CSF1R) | | 0.22 | 4.53E-07**** |  | 0.167 | 1.96E-04*** |
| TAM | CCL2 | | 0.173 | 8.32E-05**** |  | 0.127 | 4.88E-03** |
|  | CD68 | | 0.229 | 1.67E-07**** |  | 0.186 | 3.13E-05**** |
|  | IL10 | | 0.147 | 8.36E-04*** |  | 0.095 | 3.55E-02* |
| M1 macrophage | INOS (NOS2) | | 0.237 | 4.90E-08**** |  | 0.214 | 1.60E-06**** |
|  | IRF5 | | 0.095 | 3.15E-02* |  | 0.048 | 2.92E-01 |
|  | COX2 (PTGS2) | | 0.359 | 3.07E-17**** |  | 0.372 | 1.30E-17**** |
| M2 macrophage | CD163 | | 0.279 | 1.40E-10**** |  | 0.244 | 4.23E-08**** |
|  | VSIG4 | | 0.132 | 2.75E-03** |  | 0.09 | 4.52E-02* |
|  | MS4A4A | | 0.189 | 1.71E-05**** |  | 0.141 | 1.74E-03** |
| Neutrophils | CD66b (CEACAM8) | | -0.036 | 4.11E-01 |  | -0.043 | 3.39E-01 |
|  | CD11b (ITGAM) | | 0.202 | 4.11E-06**** |  | 0.151 | 7.94E-04*** |
|  | CCR7 | | 0.058 | 1.90E-01 |  | -0.043 | 3.39E-01 |
| Natural killer cell | KIR2DL1 | | 0.075 | 8.70E-02 |  | 0.047 | 2.93E-01 |
|  | KIR2DL3 | | 0.105 | 1.75E-02** |  | 0.071 | 1.17E-01 |
|  | KIR2DL4 | | 0.114 | 0.00992** |  | 0.075 | 0.0941 |
|  | KIR3DL1 | | 0.038 | 3.94E-01 |  | -0.005 | 9.07E-01 |
|  | KIR3DL2 | | 0.064 | 1.44E-01 |  | 0.033 | 4.61E-01 |
|  | KIR3DL3 | | 0.117 | 7.64E-03** |  | 0.105 | 1.97E-02* |
|  | KIR2DS4 | | 0.109 | 1.35E-02* |  | 0.083 | 0.0649 |
| Dendritic cell | HLA-DPB1 | | -0.056 | 2.04E-01 |  | -0.142 | 1.57E-03** |
|  | HLA-DQB1 | | -0.047 | 2.91E-01 |  | -0.118 | 8.88E-03** |
|  | HLA-DRA | | -0.057 | 1.99E-01 |  | -0.145 | 1.29E-03** |
|  | HLA-DPA1 | | -0.026 | 5.63E-01 |  | -0.105 | 0.0199* |
|  | BDCA-1 (CD1C) | | -0.021 | 6.29E-01 |  | -0.082 | 0.0698 |
|  | BDCA-4 (NRP1) | | 0.314 | 3.82E-13**** |  | 0.302 | 6.90E-12**** |
|  | CD11c (ITGAX) | | 0.207 | 2.21E-06**** |  | 0.145 | 1.22E-03** |
| Th1 | T-bet (TBX21) | | 0.044 | 3.16E-01 |  | -0.037 | 0.409 |
|  | STAT4 | | 0.117 | 7.78E-03** |  | 0.038 | 3.95E-01 |
|  | STAT1 | | 0.163 | 2.01E-04*** |  | 0.123 | 6.37E-03** |
|  | IFN-γ (IFNG) | | 0.012 | 0.788 |  | -0.045 | 3.22E-01 |
|  | TNF-α (TNF) | | 0.062 | 1.62E-01 |  | -0.011 | 8.02E-01 |
| Th2 | GATA3 | | 0.155 | 4.09E-04*** |  | 0.079 | 8.01E-02 |
|  | STAT6 | | 0.063 | 1.55E-01 |  | 0.078 | 8.18E-02 |
|  | STAT5A | | 0.21 | 1.49E-06**** |  | 0.152 | 7.08E-04*** |
|  | IL13 | | -0.017 | 6.94E-01 |  | -0.047 | 2.95E-01 |
| Tfh | BCL6 | | 0.204 | 3.27E-06**** |  | 0.194 | 1.43E-05**** |
|  | IL21 | | 0.057 | 1.95E-01 |  | 0.018 | 6.93E-01 |
| Th17 | STAT3 | | 0.331 | 1.34E-14**** |  | 0.347 | 2.29E-15**** |
|  | IL17A | | 0.018 | 6.79E-01 |  | -0.025 | 5.79E-01 |
| Treg | FOXP3 | | 0.207 | 2.23E-06**** |  | 0.138 | 2.13E-03** |
|  | CCR8 | | 0.252 | 7.09E-09**** |  | 0.198 | 9.73E-06**** |
|  | STAT5B | | 0.261 | 1.82E-09**** |  | 0.26 | 4.66E-09**** |
|  | TGFβ (TGFB1) | | 0.276 | 2.21E-10**** |  | 0.235 | 1.24E-07**** |
| T-cell exhaustion | PD-1 (PDCD1) | | 0.071 | 1.06E-01 |  | -0.011 | 0.801 |
|  | CTLA-4 | | 0.107 | 1.53E-02* |  | 0.022 | 6.33E-01 |
|  | LAG3 | | 0.037 | 3.98E-01 |  | -0.025 | 5.74E-01 |
|  | TIM-3 (HAVCR2) | | 0.161 | 2.38E-04*** |  | 0.092 | 4.09E-02* |

**p*<0.05; ** *p* <0.01; *** *p* <0.001; **** *P* < 0.0001
